# Recent Advances in Metal/Alloy Nano Coatings for Carbon Nanotubes Based on Electroless Plating

**DOI:** 10.3389/fchem.2021.782307

**Published:** 2022-01-06

**Authors:** Lei Zhang, Yi Chao, Kenan Yang, Daodao Xue, Shisheng Zhou

**Affiliations:** ^1^ School of Mechanical and Precision Instrument Engineering, Xi’an University of Technology, Xi’an, China; ^3^ Western Institute of Modern Vocational Education, Xianyang, China; ^2^ Shaanxi Polytechnic Institute, Xianyang, China; ^4^ Faculty of Printing, Packaging Engineering and Digital Media Technology, Xi’an University of Technology, Xi’an, China; ^5^ Shaanxi Provincial Key Laboratory of Printing and Packaging Engineering, Xi’an University of Technology, Xi’an, China

**Keywords:** carbon nanotubes, electroless plating, meta, alloy, nano coatings

## Abstract

A large number of researches on the electroless plating of carbon nanotubes and their applications after plating have emerged, which has attracted more and more attention. In this review article, the existing electroless plating methods for carbon nanotubes were briefly summarized, and the surface coatings were listed and analyzed in detail. At last, the related applications after electroless metal/alloy coatings of carbon nanotubes were discussed in detail. This study aims to provide a reference for the research and improvement of different electroless metals/alloys coatings of carbon nanotubes. After a clear understanding of the electroless metal/alloy coatings of carbon nanotubes, the appropriate coating can be selected according to the actual situation, so that the carbon nanotubes after plating can be used as reinforcement and modification materials for better satisfaction of the needs, and the application of plated carbon nanotubes has reference significance in more fields.

## 1 Introduction

### 1.1 Preamble

Carbon nanotubes (CNTs), as quasi one-dimensional nanomaterials, have been widely studied and applied since their discovery due to their excellent mechanical properties and special electrochemical, thermal and magnetic properties (Li et al., 2009). They have been widely used in interconnects (Ngo et al., 2007; Desmaris et al., 2014), energy storage (Nihei et al., 2005), transistors (Martel et al., 1998; Tans et al., 1998), touch screens (Cao et al., 2015), membrane switches and other conductive and thermally conductive materials (Han et al., 2012). These capabilities are due in large part to their extremely small size (about 1–10 nm in diameter), high aspect ratio (>1,000), high structural and chemical stability (Liu et al., 2007). However, it has been found that for some applications where metallic properties (e.g., interconnects), conductivity, thermal conductivity, and magnetism are required, the use of pure CNTs as additive materials has major limitations. As pristine CNTs have low chemical activity, large degree of entanglement, and insignificant magnetic properties, if they are directly added to metal matrix composites, they will easily agglomerate due to poor wettability and weak interfacial bonding between them and the matrix material, which will not play a proper role in strengthening the matrix material but reduce the physical and mechanical properties of the material (Zhang et al., 2015). Accordingly, it is necessary to modify CNTs to better exploit their advantages in matrix materials. Electroplating has been proved successful in fabricating film/bulk composite with good mate rial properties but not suitable for individual hybrid metal/CNT structure. Electroless plating could be a good method to fabricate individually separated metal/CNT nanowire owing to its unique deposition process (Cha et al., 2005; Peng and Chen, 2009). Electroless plating is a simple, energy-saving and environmentally friendly electroless deposition technology. It refers to the reduction of metal ions in the plating solution to metal and deposits in the active center of the material to be plated through a controllable redox reaction with a suitable reducing agent under the condition of no external current (Maqbool et al., 2013; Jagannatham and Haridoss, 2015). The electroless plating method has the characteristics of simple process, easy operation, uniform coating, low porosity, and good wear resistance. This process does not require an external power supply during the preparation process, and the materials immersed in the plating solution can be evenly covered by the plating layer. Therefore, this technology has received widespread attention. For example, the modification of wood, fibrils (Hui et al., 2020; Chen et al., 2021) often uses electroless plating. Due to its wide range of use, high quality of plating, and the ability to obtain a complete and uniform metal/alloy coatings on the surface of metallic or nonmetallic materials of any shape. Its appearance provides a convenient way for the successful modification of carbon nanotubes. Although different from the unique anisotropic multi-channel structure characteristics of easy electroless plating of wood (Hui et al., 2021), carbon nanotubes have a large aspect ratio and a large specific surface area, excellent mechanical properties and special electrochemical, thermal and magnetic properties. Increasing the activity of carbon nanotubes through plating has a greater strengthening effect on the material in terms of mechanics, electrical conductivity, thermal conductivity, tribology, and corrosion resistance. So it can be used as a filler to be more widely used in metal matrix composite materials, magnetic composite materials, electrically and thermally conductive materials, energy storage materials and catalytic materials, etc. A large number of studies have found that high-quality metal/alloy coatings have been successfully plated on the surface of pretreated carbon nanotubes by controlling the plating conditions (Chen et al., 2000; Chen et al., 2002).

At present, the common coatings on the surface of CNTs include silver, copper, nickel and alloys (such as Ni-P, Ni-Co, Ni-Co-P, etc.), while gold and cobalt coatings are relatively rare. Plating combines the characteristics of the metal/alloy of the coating with the characteristics of the CNTs themselves, thereby imparting stronger or new properties to the CNTs (Elbasuney et al., 2019). It has been found that the coating increases the activity of CNTs, improves their physical and mechanical properties, and provides good wettability and interfacial bonding between them and the matrix material, which can be used as fillers in a wider range of applications such as metal matrix composites, magnetic composites, electrically and thermally conductive materials, energy storage materials, and catalytic materials, which have a greater strengthening effect on materials in terms of mechanics, electrical conductivity, thermal conductivity, tribology, and corrosion resistance (Esawi et al., 2009).

### 1.2 Synopsis

Following this introduction section, this review will, in Section 2, make a brief discussion of fundamental aspects of underlying electroless plating processes. In Section 3, this study will elaborate the classification of electroless plating metal alloy plating and its physical and mechanical properties after electroless plating. Section 4 details the application of carbon nanotubes after electroless plating. Finally, in Section 5, the review ends with conclusion and future prospect.

## 2 Methods for Electroless Plating of Carbon Nanotubes

At present, there are two technological methods for electroless plating of CNTs, namely, the traditional direct coating with mixed aqueous solution and the new ultrasonic spray atomization assisted electroless plating (EPUSA). The methods for electroless plating of CNTs will be analyzed and compared in detail below.

### 2.1 Traditional Method

The traditional electroless plating is carried out directly in the form of mixed aqueous solution. Usually, the reducing agent and the metal salt solution in the plating bath are directly mixed in a certain proportion or added dropwise (Zhao et al., 2017). However, it takes a certain amount of time to diffuse and homogenize the components in the plating solution, which will cause spatial inhomogeneity in the entire system, and the metal salt, reduced agent and the failed uniform mixing of CNTs. In addition, CNTs will agglomerate in the plating solution, and the overall quality of the resulting plating layer is not high.

### 2.2 Ultrasonic Spray Atomization Method (EPUSA)

In the EPUSA method, the suspension of the metal salt solution and the reducing agent-CNTs were ultrasonically atomized into droplet form; then the two were brought into contact in the reaction vessel and the metal reaction was completed. Since each initially contained reducing agent-CNTs or metal ions was relatively limited, it could be ensured that two or more simultaneous reduction reactions occurred when contacting, so that the reduced metal nanoparticles were deposited on the active sites on the surface of the CNTs. This process was realized within micrometer scale droplets generated by ultrasonic spray atomization, for the purpose of getting higher uniformity of silver deposits/coatings on MWCNTs (Choi et al., 2014).

## 3 Classification of Electroless Plating on the Surface of Carbon Nanotubes

### 3.1 Carbon Nanotubes Electroless Gold Plating

Gold has excellent corrosion resistance, good electrical conductivity, thermal conductivity and processability, so it can be used in electronics, communications technology, chemical technology and many other fields (Dondoi et al., 2006; Yang et al., 2014). However, as a precious metal, gold is very expensive, which largely limits its application. CNTs are very economical and have unique physical and mechanical properties, which have successfully attracted the wide attention. Researchers have combined gold with CNTs and coated them with a layer of gold nanoparticles, which not only retains the excellent properties of gold but also increases the cost-effectiveness of the coating.

**FIGURE 1:**
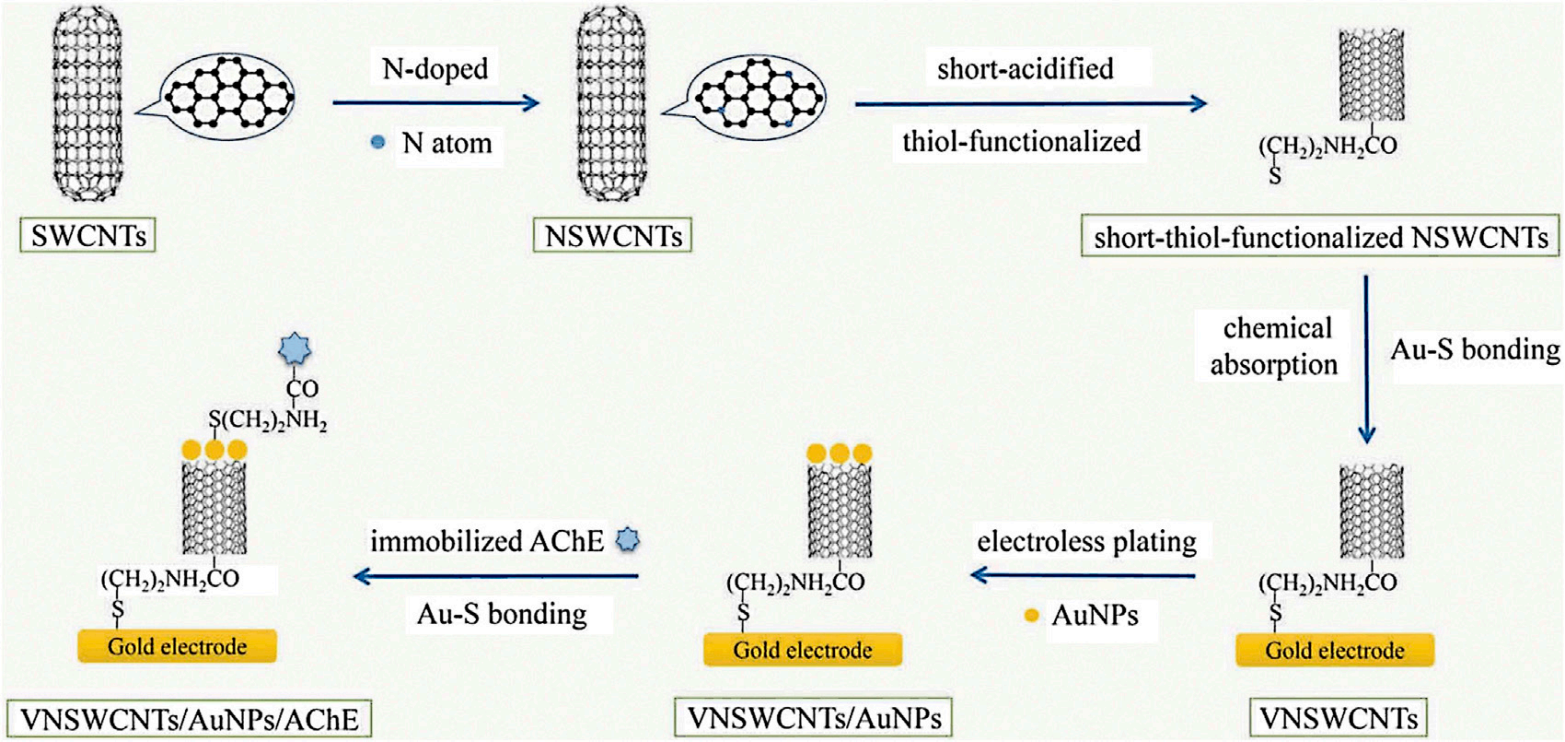
The construction of the AChE biosensor (Xu et al., 2019) (published in Int J Environ Anal Chem 2019).

Xu et al. constructed an acetylcholinesterase (AChE) biosensor based on gold nanoparticles (AuNPs) chemically plated on vertical nitrogen-doped single-walled CNTs (VNSWCNTs) (the schematic diagram for the construction process is shown in Figure 1). The modified gold electrode was soaked in 2 ml distilled water contained 5 μL of 50 mM HAuCl4 in HCl for 30 min. This was followed by rapid addition of 15 μL of 50 mM reducing agent NaBH4 in NaOH to the solution in the reagent bottle, then the colour was slightly lavender (Martin et al., 2010; Chaudhari et al., 2016). After standing for 30 min, the modified gold electrode was taken out to obtain an Au/VNSWCNTs/AuNPs modified electrode. Finally, this electrode was immersed in 5 mM NH2–(CH2)2–SH solution for 4 h, and the AuNPs were attached to NH2–(CH2)2–SH *via* Au–S bonding (Ju et al., 2015). The modified electrode was then coated with 10 μL (2 U/mL) AChE; after that, the amino group at the other end of NH2–(CH2)2–SH cross-linked with carboxyl bond of AChE. The AuNPs constructed by this method are considered to be more stable than the covalent self-assembled AuNPs. Due to the synergistic effect between AuNPs and VNSWCNT, the AChE biosensor prepared by this method has excellent electron transport capability, and it performs better than the biosensor in previous studies in terms of stability, sensitivity and durability (Xu et al., 2019). Besides, Feng et al. prepared the sulfhydryl-modified carbon nanotubes groups by reacting the pretreated carbon nanotubes with LiAlH4, PBr3 and NaHS in turn, and then placed them in an electroless plating solution to prepare quasi-one-dimensional gold nanowires with an average diameter of less than 50 nm. Experiments have proved that the formation mechanism of gold nanowires is that in the presence of sulfhydryl groups (-SH), gold nanoparticles self-assemble on the surface of carbon nanotubes (CNTs) (Feng, 2008).

### 3.2 Carbon Nanotubes Electroless Cobalt Plating

Due to the superior magnetic properties, cobalt is widely used in the manufacture of high-performance magnetic materials. Magnetic materials are important functional materials, and they play a very important role in the electronics industry and high-tech fields (Guo et al., 2006; Turtelli et al., 2006; Ruitao and Kang, 2008; Hyie et al., 2017). Electroless plating was utilized to deposit Cobalt (Co.) on the surface of multi-walled carbon nanotubes (MWCNTs), the Co-MWCNTs composites showed a higher impedance which implies a better potential absorbing property and makes Co-MWCNTs probable to be utilized in electromagnetic absorbing field (Bao et al., 2011). By chemically coating CNTs with cobalt to give them magnetic properties, they are widely used as magnetic reinforcements for composite materials.

After the pretreated carbon nanotubes are electrolessly plated with cobalt under a suitable process, it is found that the surface is uniformly and continuously covered with a layer of centered cubic nanocrystalline cobalt coating. After metallization, the carbon nanotubes still keep the original fibrous form, and the thickness of the resulting coating is about 5–15 nm. Studies have shown that changes in the pH value and plating time during the electroless plating process have a great influence on the thickness and uniformity of the coating (Wilson et al., 2011). After cobalt coating, the magnetic properties of carbon nanotubes are greatly improved. It was found that the saturation magnetization strength of Co-MWCNTs was 2.81 emu/g and the coercivity was about 4 times higher than that of cobalt powder (308Oe) after chemical cobalt plating. Bao et al., explored the effect of metal salt concentration and pH value change on the quality of the plating during the chemical cobalt plating process. It was found that the electromagnetic properties of Co-MWCNTs composites were better than those of MWCNTs composites, and the chemical cobalt plating process improved the magnetic properties of the carbon nanotube composites, and calculations based on the measured parameters showed that the cobalt-plated carbon nanotube composites improved the microwave absorption capacity of the carbon nanotube composites (Bao et al., 2011). The Co-MWNT composites have lower surface reflection, which have the potential to be applied as the matching part in two-layer absorbing material.

### 3.3 Carbon Nanotubes Electroless Silver Plating

Silver has excellent ductility, electrical and thermal conductivity, and it is widely used in electrical and electronic materials, photographic materials, chemical materials, etc. The deposition of silver on CNTs allows for combination or enhancement of properties, and has been extensively studied in applications such as field emission, polymer-reinforced fillers, and composite materials (Pal and Sharma, 2015). The formation of silver plating on the surface of CNTs is achieved by the reduction of silver ions deposited on their active sites by reducing agents. With the appropriate increase of plating time, the reduced silver nanoparticles grow up along the nucleation center in the normal and tangential directions and gradually cover the whole surface of CNTs, making the density of CNTs increase and the resistivity decrease after plating (Feng and Yuan 2004). Youngseok et al. have developed a novel silver/conducting polymer composite by the incorporation of CNTs, resulting in a significant increase in electrical conductivity. The interfacial contact was improved by the electroless plating of silver on the CNTs (Oh et al., 2008).

#### 3.3.1 Physical Properties of Silver-Plated Carbon Nanotubes

The deposition uniformity and microstructure characteristics of the silver coating on CNTs are important physical characteristics after silver plating (Daoush et al., 2009). Numerous studies have found that the more uniform the carbon nanotubes are dispersed during electroless silver plating, the more uniform the coating morphology will be. Conversely, the more uniform the silver coating, the less likely the carbon nanotubes agglomerate during the plating process, and the thickness; continuity of the coating uniformity can also be controlled by plating conditions. The silver coating on the surface of carbon nanotubes is formed by reducing and depositing silver ions on the active sites by a reducing agent. With the appropriate increase of the plating time, the reduced silver nanoparticles move toward the normal and cut along the nucleation center. It grows up and gradually covers the entire surface of the carbon nanotubes, so that the density of the carbon nanotubes increases after plating, and the resistivity decreases (Feng et al., 2005).

#### 3.3.2 Mechanical Properties of Silver-Plated Carbon Nanotubes

The original CNTs are soft and highly entangled. The silver-plated CNTs integrate the advantages of metal and CNTs, so that the hardness and strength of the CNTs are greatly improved, and the surface contact area is increased. As a result, the friction resistance under certain loads is significantly improved compared to uncoated CNTs, making it easier to use silver-plated CNTs in microsystems with moving components (Zhang and Liu, 2016; Zhang et al., 2017; Yang et al., 2018).

#### 3.3.3 Other Properties of Silver-Plated Carbon Nanotubes

CNTs themselves have high thermal conductivity in the axial direction but very low thermal conductivity across the axial direction. The thermal conductivity will be further increased after silver plating, because metallic silver itself has good thermal conductivity. With the tremendous increase in the power and packaging density of electronic devices in recent years, thermal management and thermal interface materials (TIM) have become more and more important.

### 3.4 Carbon Nanotubes Electroless Copper Plating

Copper is abundant in nature, and it has excellent electrical conductivity, thermal conductivity, ductility, corrosion resistance, wear resistance, etc. Copper is widely used in the fields of power electronics, machinery and metallurgy, transportation, light industry, new industries, and high technology (Sahraei, 2017). The surface state and structure of CNTs can be improved by electroless copper plating, so that they not only exhibit high thermal and chemical stability, but also improve their dispersibility and wettability with the substrate.

#### 3.4.1 Physical Properties of Copper-Plated Carbon Nanotubes

Under a suitable electroless copper plating process, through scanning electron microscopy and transmission electron microscopy, it is found that a layer of centered cubic nanocrystalline copper coating is uniformly and continuously covered on the surface of the CNTs. The metalized CNTs are still intact and uniform (Arai and Endo, 2003; Yamagishi, 2003; Araia et al., 2004). In addition, compared with unplated CNTs, the copper-plated CNTs have increased density and decreased resistivity, and it has good dispersion uniformity in deionized water.

#### 3.4.2 Mechanical Properties of Copper -Plated Carbon Nanotubes

The surface modification metallization of CNTs with copper not only increases their surface active centers and improves the bonding of nanotubes with resins or ceramics, but also maintains the excellent properties of CNTs in composites, which greatly expands the application fields of CNTs (Wang, 2011; Wang, 2014). After sintering with metal powder (such as copper powder) as composite material, the strength of the composite material increases due to the strengthening of grain boundaries because the CNTs can prevent the growth of copper particles during the sintering process, which increases the number of non-metallic interfaces between copper particles (Han and Kim, 2013).

#### 3.4.3 Other Properties of Copper -Plated Carbon Nanotubes

Due to the high thermal conductivity (TC) and low thermal expansion coefficient, CNTs are considered as potential enhancers for copper-based thermal management materials. The sp2 hybridized carbon atoms in CNTs lead to poor wettability between CNTs and Cu, which results in weaker interfacial bonding. Therefore, in order to better utilize the excellent thermal conductivity of CNTs and increase their activity to improve the wettability, a layer of metallic Cu was coated on the surface of CNTs (Kim, 2008). Cu itself has good thermal conductivity, and the combination of the two can not only further increase the TC of the composite, but also sufficiently improve the inter-facial bonding between CNTs and the metal matrix.

### 3.5 Carbon Nanotubes Electroless Nickel Plating

Nickel metal has properties such as high hardness, good toughness and excellent ferromagnetism, and it is often doped in other metals or non-metals as a second phase. CNTs are widely used as reinforcement materials in the field of composite materials. In order to make fuller use of CNTs’ superior properties, the surface modification, modification and coating are often applied to CNTs. Using the chemical nickel plating process, the continuous high-strength bonded nickel plating can be deposited on the surface of CNTs, which can greatly broaden the application fields of CNTs. Therefore, the study of chemical nickel plating on CNTs has attracted great interest from researchers (Yu and Shen, 2009).

#### 3.5.1 Physical Properties of Nickel-Plated Carbon Nanotubes

The pretreated CNTs are chemically active and hydrophilic, which can attract the metallic nickel particles to its surface. On the contrary, if there is no pretreatment before chemical plating, the metallic nickel particles will not be adsorbed on the surface of CNTs. With the appropriate increase in the deposition of nickel, the coating tends to be uniform and continuous, and there is a firm bond between metallic nickel and CNTs. Since metallic nickel is inherently well ferromagnetic, the coating CNTs with nickel makes them easy to handle with magnetic forces. Accordingly, the study on how nickel-plated CNTs respond to external magnetic fields is important for their application as fillers for magnetic materials. In addition, deposit uniformity is an important physical property and a significant advantage of the electroless nickel process. It is the ability to produce uniform thickness on parts with complex geometries and shapes. The current density effect typically associated with electroplating is not a factor here; so sharp edges, deep recesses and blind holes are readily plated to have uniform thickness with electroless nickel process. Electrodeposition leads to the excessive buildup at projections, and the edges and finish-grinding operation may be required. In such a context, electroless deposits avoid these drawbacks (Jothi et al., 2013).

#### 3.5.2 Mechanical Properties of Nickel-Plated Carbon Nanotubes

Nickel plating of CNTs provides significant improvements in mechanical properties and reliability as well as plating of other metals. Usually, we choose suitable plated CNTs to be doped into composite materials according to different applications. As modern electronic devices require higher performance and increasingly smaller sizes, the solder joints of components in electronic packaging have become a key consideration, compared to undoped lead-free solder in which nickel-plated CNTs are doped into the Sn-58Bi solder alloy as a second phase, the nickel-plated CNTs not only enable the solder joints to cure at lower temperatures, but also have greatly improved shear strength, ductility and durability improved. The addition of nickel-plated CNTs significantly improves the performance of the composite solder because of the good mechanical and electrical properties of the CNTs themselves, and the addition of nickel metal further enhances the mechanical properties such as hardness, wear resistance and ductility (Zhang et al., 2008).

#### 3.5.3 Other Properties of Nickel-Plated Carbon Nanotubes

Primitive CNTs have good thermal conductivity and flexibility, and CNTs are usually considered as the reinforcing phase to improve the thermal conductivity and toughness of composite materials, but the direct addition of CNTs to composite materials will cause them to aggregate under the action of van der Waals forces, making it difficult to effectively disperse in the matrix material. Electroless nickel plating can not only make CNTs uniformly distributed in the composite material but also improve its thermal conductivity, and the presence of nickel plating facilitates the application of CNTs in thermal management materials (Xiong et al., 2006).

### 3.6 Carbon Nanotubes Electroless Alloy Plating

Alloys have higher strength, hardness, and corrosion resistance than single metals, and can also give CNTs composites excellent properties compared to monometallic nanoparticles coated on CNTs. They have shown significant improvements in aspects such as thermal conductivity, electrical conductivity, magnetic properties, catalytic properties, corrosion resistance, and wear resistance, so they are even better than monometallic nanoparticles. The formation of composite coatings on CNTs and the effect of composite coatings on the properties of carbon nanotube composites have been extensively investigated. At present, the main alloy coatings coated on CNTs are Ni-Co alloy, Ni-P alloy, Ni-B alloy, Co-B alloy, Ni-Co-P alloy, Ni-Co-B-P alloy, Fe-Co alloy, Ni-Cu-P alloy, Pd-Ni alloy, Sn-Cu alloy, and Ni-Pd-Sn alloy (Zhao et al., 2017). The properties given to CNTs vary because of the different alloys coated. Electroless deposited alloy features and types of metallic non-electrolytic alloy coatings are summarized in Table 1.

**TABLE 1:** Features and types of electroless metallic alloy coatings.

Use	Alloy types
Corrosion protection	Ni-P, Ni-P-Mo, Ni-Sn-P, Co-P, Ni-Cu-P
Wear resistance	Ni-B, Ni-B-Mo, Ni-B-Sn, Co-P, Co-P-W
Magnetic	Au-Ni, Au-Co; Ni-Co-P, Ni-Co-B, Ni-Fe-P
Solderability	Sn-Pb, Ni-P
High temperature	Co-W-B, Ni-Re-P
Diffusion barrier	Ni-P

#### 3.6.1 Physical Characteristics of Alloy-Plated Carbon Nanotubes

Like other monometallic alloys, CNTs can be coated with uniform, dense and continuous layers under appropriate coating conditions. For some heat-treated alloys, different phase transformation processes occur at different temperatures. When the heat treatment temperature is low, the alloy plating shows both amorphous and crystalline states, and as the heat treatment temperature rises, a complete crystalline structure is obtained, in which the grain size increases significantly with the increase of temperature. When Ni-Co-P alloy, Fe-Co alloy, Ni-P alloy and Co-P alloy are electrolessly plated on the surface of CNTs, the coating thickness is about 5–20 nm. The saturation magnetization, coercive force and microwave absorption characteristics of the tube are quite different, so it is very important to explore the appropriate alloy concentration ratio for the magnetic performance (Pang et al., 2011).

#### 3.6.2 Mechanical Properties of Alloy-Plated Carbon Nanotubes

After the post-plating heat treatment process, the microhardness of the coating is greatly improved. With the moderate increase of MWCNTs concentration in the chemical plating solution, the microhardness gradually increases. The heat treatment not only reduces cracks and pores of the coating but also has the effect of grain refinement, which makes the coating become more dense and continuous, and the microhardness and strength becomes higher. The alloy-plated CNTs also showed significant improvement in ductility, stiffness, frictional properties and wear resistance, and it was found that the concentration of MWCNTs had an effect on the frictional properties and wear resistance of the coating, and the friction coefficient of the coating gradually decreased with the increase of MWCNTs concentration (Chung, 2012).

#### 3.6.3 Other Properties of Alloy-Plated Carbon Nanotubes

CNTs electroplated with Ni-P coatings are excellent in terms of corrosion resistance, which increases after heat treatment, implying that the coating structure becomes denser and more homogeneous after heat treatment. Because of their high electrical and thermal conductivity, CNTs are often used as fillers to enhance the thermal conductivity of polymer composites. contact each other to form a thermal path.

## 4 Application of Carbon Nanotubes After Electroless Plating

### 4.1 Magnetic Composite

In order to obtain better efficiency in electromagnetic wave shielding, low density, designable and dimensionally stable polymeric magnetic composites have been proposed and developed. Polymer magnetic composites usually consist of two parts—a polymer that provides good compatibility and a magnetic filler, as well as the inorganic magnetic nanoparticles such as nickel, cobalt or alloys related to nickel-cobalt. CNTs are common fillers due to their excellent mechanical, thermal, and electrical properties, but unmodified CNTs cannot obtain excellent magnetic properties due to their small maximum saturation magnetization intensity. In order to optimize the performance of CNTs as magnetic fillers, they must be coated with magnetic substances to modify them. Nickel and cobalt powders are typical magnetic fillers, with high conductivity and high saturation magnetization, but with high density and high cost. Plated nickel/cobalt multi-walled CNTs obtained by chemical plating method for comprehensive cost will become magnetic fillers with high magnetic properties and medium cost (Gopiraman et al., 2013).

Jagannatham et al. electroless nickel plating on multi-walled carbon nanotubes (MWCNTs) synthesized by arc discharge method, and then discussed the influence of plating time on the morphology and magnetic properties of the coating (Jagannatham et al., 2015). Zhang et al. obtained nickel-plated multi-walled CNTs (Ni/MWCNTs) by chemical plating, and then prepared a novel polymeric magnetic composite based on thermoplastic acrylate pressure-sensitive adhesive (PSA) using Ni/MWCNTs as filler, and the results of vibrating sample magnetometer showed that the increase of Ni/MWCNTs content could improve the saturation (Zhang et al., 2012). Liang et al. chemically coated CNTs with Co-P alloy and found that the saturation magnetization intensity of homogeneous Co2P-CNTs nanocomposites was 2.81 emu g-1 (Liang et al., 2016). Wang et al. coated Fe-Co alloy nanoparticles onto Fe-filled CNTs by chemical plating and evaluated the microwave absorption properties of the samples, and the results showed that coating Fe-Co alloy nanoparticles followed by heat treatment can improve the soft magnetic properties of Fe-filled CNTs, resulting in more effective microwave absorption (Chen et al., 2008).

### 4.2 Mechanical and Frictional Performance Improvement Materials

CNTs have become one of the most promising materials in the electronic field in the future due to their excellent mechanical properties, friction properties, and electrical and thermal conductivity. They have been widely used in interconnections, transistors, and thermal and electrical materials. However, for some applications where metal properties are particularly important (such as interconnection) and occasions that require high device reliability and electrical performance, pure CNTs have great limitations. A large number of studies have confirmed that the addition of plated CNTs can significantly improve the mechanics and tribology of composite materials (Choudary et al., 2001; Barlian et al., 2009; Zhao et al., 2015).

**FIGURE 2:**
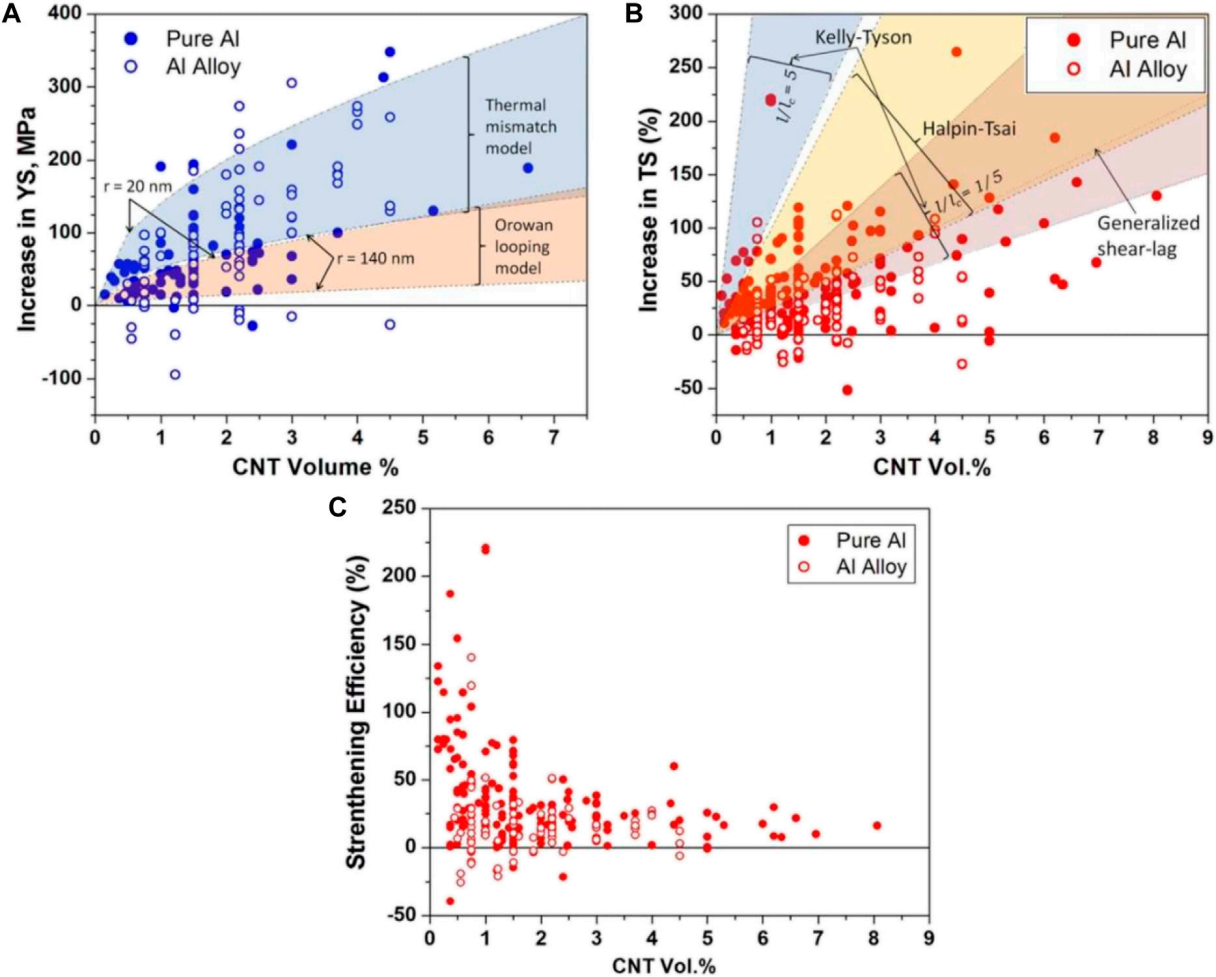
Plots showing variation of tensile properties of Al-CNT composites as a function of CNT content. **(A)** Increase in the YS, **(B)** increase in TS and **(C)** Strengthening efficiency (Jagannatham et al., 2020) (published in Carbon 2020).

Ma et al. blended acrylate resin with Ag-CNTs and Ag-GNs to synthesize a new conductive adhesive (ECA) and found that the conductivity reached 8.71 S/cm and the shear strength was 0.47 MPa when the concentration of the composite filler was 30 wt% (21 wt% of Ag-GNs and 9 wt% of Ag-CNTs) (Huan et al., 2015). Zhao et al. prepared CNTs-Ag composites by solution ball milling (SBM) of silver-plated CNTs (Ag-CNTs) obtained by plain chemical plating (CEP) and ultrasonic spray atomization (EPUSA), respectively, with Ag powder, followed by densification by spark plasma sintering (SPS). It was characterized that the composites made by EPUSA and SBM processes had better mechanical and electrical properties compared with CEP and SBM processes (Zhao et al., 2018). M. Jagannatham and Adnan Maqbool et al. prepared Cu-CNTs reinforced Al matrix composites and compared with pure Al-CNT composite materials, the yield strength and tensile strength of Al alloy-CNT composite materials has increased more. This can be attributed to the fact that most of the aluminum alloys used are precipitation hardening, and the addition of CNT improves the precipitation hardening characteristics. The percentage increase in the tensile strength is high for pure Al-CNT composites compared to Al alloy-CNT composites. Because compared with aluminum alloy-carbon nanotube composite material, pure aluminum carbon nanotube has a lower porosity. Figure 2A shows the increase in the Yield Strength (YS) of the composites as a function of CNT content. Figure 2B shows the increase in Tensile Strength (TS) of the composites with CNT content. Figure 2C shows the strengthening efficiency of CNT in Al-CNT composites as a function of CNT content (Jagannatham et al., 2020).

### 4.3 Catalytic Material

With high surface area, porous structure and high catalytic capacity, multi-walled CNTs have been widely used in high-energy systems. MWCNTs can enhance the heat transfer during high-energy combustion reactions, and the gaseous products are easily absorbed by the catalyst active sites, so they are favorable for catalytic reactions. MWCNTs are excellent carriers for common combustion rate catalysts (Yong et al., 2018). The obtained Cu-MWCNTs were annealed at 250°C to obtain CuO-MWCNTs with good catalytic ability by Sherif Elbasuney et al. The synthesized CuO-MWCNTs were then encapsulated into ammonium perchlorate (APC) oxidant by solvent-anti-solvent technique, and differential scanning calorimetry (DSC) and thermogravimetric analysis (TGA) were used to study the catalytic performance of CuO -MWCNTs on the decomposition of APC by differential scanning calorimetry (DSC) and thermogravimetric analysis (TGA), and the results showed that 1 wt% of CuO-MWCNTs reduced the heat absorption decomposition of APC by 16.3%, and the original two exothermic decomposition stages were combined into one stage with a 100% surge in total heat release (Sherif et al., 2019). Liu et al. successfully synthesized Ni-B-coated multi-walled CNTs (MWCNTs) by a chemical deposition process. The catalytic activity of MWCNTs/Ni-B nanoparticles was evaluated and it was found that the catalytic hydrogenation by MWCNTs/Ni-B nanoparticles resulted in the selective conversion of styrene to ethylbenzene. The highest conversion of 99.8% was achieved under appropriate reaction conditions, which indicates the high catalytic activity of MWCNTs/Ni-B nanoparticles (Liu et al., 2011). Wu et al. grew CNFs on CNTs by chemically plating Ni-P alloy in order to synthesize Ni-P/CNT-CNFs composites, which were directly used as electrocatalysts in methanol-resistant redox reactions (ORR) (Wu et al., 2017). Experiments showed that this irregularly oriented hybrid material, in addition to being an ideal candidate for non-precious metal electrocatalyst carriers, exhibited satisfactory ORR activity and excellent methanol tolerance in alkaline solutions. This study provides a promising option for the synthesis of non-precious metal catalysts for fuel cells.

### 4.4 Electrically and Thermally Conductive Materials

In recent years, the electronics industry has developed rapidly. Electronic equipment not only tends to be miniaturized, lightweight, and highly functional, but also power and packaging density are gradually increasing. Therefore, it is becoming more and more important to obtain materials with good electrical and thermal conductivity. CNTs have a high aspect ratio and excellent electrical and thermal conductivity, and can be used as potential enhancers in electrical and thermal conductivity composite materials. However, the wettability between unmodified CNTs and metal-based materials is poor, and the interface the weak bonding is not conducive to its excellent performance. Therefore, CNTs must be modified to meet application requirements. The success of electroless plating provides an effective way to solve this problem. Zhang et al. prepared Ag surface-modified CNTs and then mixed epoxy resin and nanotubes to obtain thermal interface materials (TIM), and the conductivity of epoxy-Ag-coated CNTs was analyzed to be much higher than that of epoxy-uncoated CNTs (Zhang et al., 2012). Choi et al. investigated the effects of nickel-plated MWCNTs and uncoated MWCNTs on the thermal conductivity and fracture toughness of alumina-reinforced epoxy composites, and the experimental results showed that the Ni-MWCNTs/Al2O3/epoxy composites had better thermal conductivity and fracture toughness than the MWCNTs/Al2O3/epoxy composites (Choi et al., 2013). Chen et al. investigated the effect of copper-plated CNTs on the microstructure and thermal conductivity (TC) of copper matrix composites (Chen et al., 2017). The results showed that the CNTs modified by copper nanoparticles were uniformly dispersed and embedded in the copper matrix, and the uniform dispersion of CNTs and the reduction of interfacial thermal resistance led to the improvement of the thermal conductivity of the carbon nanotube-copper composites.

**FIGURE 3:**
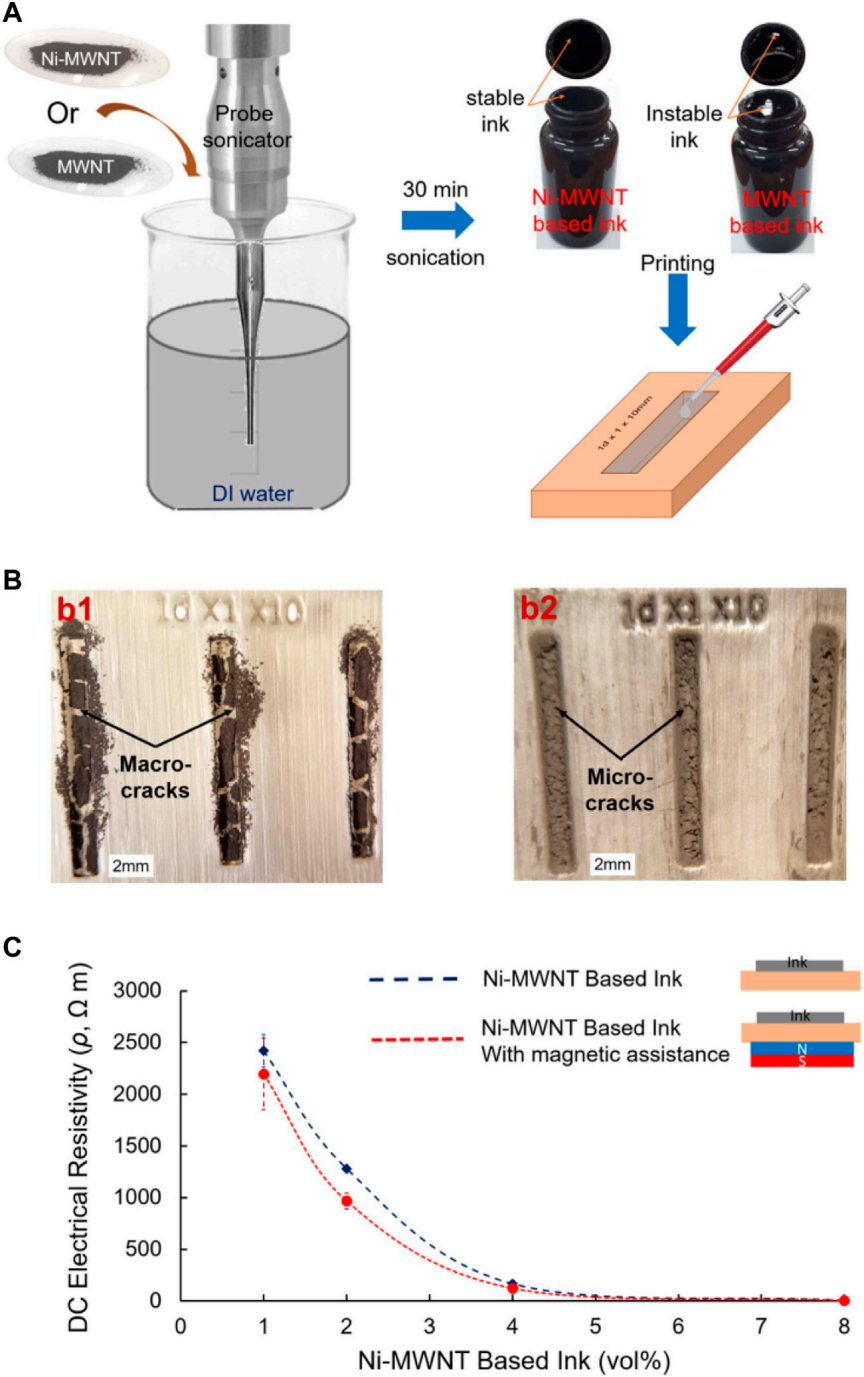
Water-based conductive inks. **(A)** The procedure for ink preparation. Ni-MWCNTs or MWCNTs are dispersed in DI water using a probe sonicator and the ink poured into a plastic template. **(B)** Post drying, optical images show the morphology of the printed lines created by either MWCNT or Ni-MWCNT based inks (2 vol%). Macroscale cracks are clearly observed in the case of MWCNTs, while smaller microscale cracks are observed for the other case. **(C)** The measured electric resistivity of the lines printed by Ni-MWCNT based inks for different volume loadings with and without magnetic assistance (Ahmed et al., 2016) (published in Mater. Res. Express 2016).

The plated CNTs are also used for the preparation of conductive pastes in the field of printed electronics due to their excellent electrical conductivity. Ahmed M Abdalla et al. chemically deposited magnetic nickel nanoparticles on multi-walled CNTs (MWCNTs) to produce Ni-MWCNT hybrids (NiCH), which are electrically conductive and have high magnetization and elastic modulus, and prepared Ni-MWCNT macrostructures with controlled morphology by applying a strong magnetic field with NiCH, and these macrostructures can be used for nanoscale and micron-scale filtration as well as printed circuits. Since the presence of nickel plating layer makes Ni-MWCNT hydrophilic, different concentrations of hydrophilic Ni-MWCNT samples and hydrophobic MWCNT samples were further dispersed in water to synthesize aqueous conductive inks as shown in Figure 3. The results showed that Ni-MWCNTs with 8% volume concentration had the lowest resistivity of 5.9 Ωm for printed circuits. compared with hydrophobic MWCNT inks, the degree of agglomeration of the dried hydrophilic Ni-MWCNT ink was low, and the cracks produced by the printed lines were very small. In addition, printing the Ni-MWCNT circuit with the aid of a magnetic field further reduces the resistivity. The following figure shows the preparation of water-based conductive ink, the morphology of the printed circuit after drying, and the resistivity of the Ni-MWCNT ink at different volume concentrations (Ahmed et al., 2016).

**FIGURE 4:**
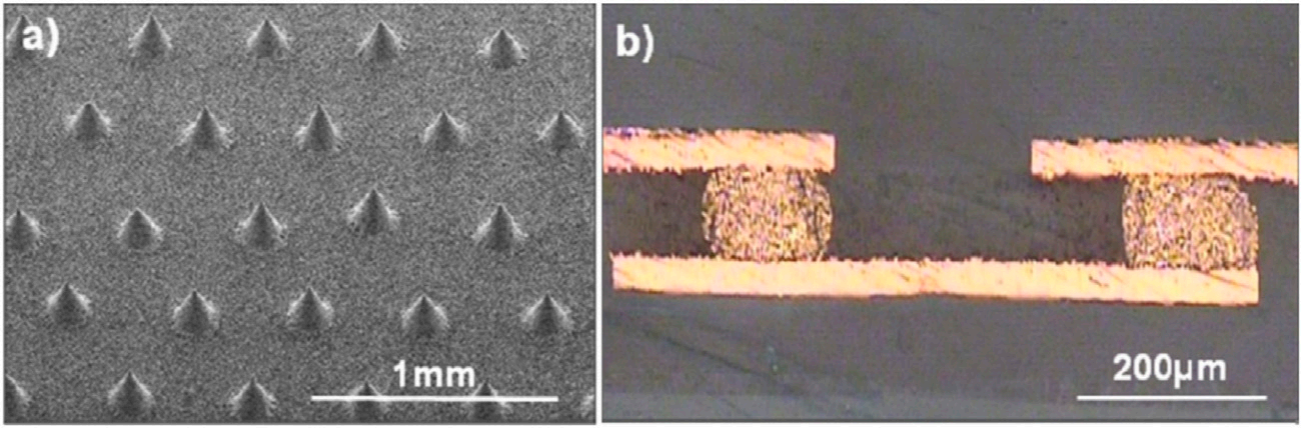
Bump interconnects formed by the Ag-plated SWCNT–Ag paste: **(A)** Screen-printed conductive bumps and **(B)** a cross-sectional view of bumps connecting copper foils after the photolithographic patterning (Oh et al., 2008) (published in Nanotech. 2008).

Youngseok Oh et al. developed a new type of silver/conductive polymer composite by combining silver-plated single-walled CNTs (SWCNT-Ag) (Oh et al., 2008). A conical conductive bump with a diameter of 130 μm at the bottom and a height of 185 μm, as shown in Figure 4A, was printed on the composite material using screen printing to demonstrate the performance of the material as a multilayer printed circuit board electrical interconnect, and the resistance of the SWCNT-Ag bump was measured to be 3.2 mΩ, which is 83% lower than that of commercial silver pastes on the market. The resistance of this SWCNT-Ag paste is 83% lower than that of commercial silver pastes in the market. The cross-sectional view of the connected copper foil bump is shown in Figure 4B, and it can be seen that the electrical signal transmission of the bump is good. Thus, it is demonstrated that SWCNT-Ag paste has excellent printability as electrical interconnects.

## 5 Conclusion

In this paper, a brief classification and summary of carbon nanotube electroless plating methods was conductedm and a detailed classification and summary of carbon nanotube electroless metal/alloy nano-coating were carried out, which can be used for reference for the future research and improvement of the electroless plating of CNTs. In addition, the application progress of electroless metal/alloy nano-coating of carbon nanotubes in aspects such as electrical, mechanical, thermal, tribological, corrosion resistance and magnetic properties were discussed in detail. At present, although electroless CNTs have been widely investigated and applied, it is essential to have a good understanding of its electroless plating surface coatings firstly, so as to better take advantage of electroless plating CNTs. Therefore, it is critical to study the electroless metal/alloy nano-coating of carbon nanotubes for obtaining plated CNTs with good appearance quality and excellent comprehensive performance.
